# Tumor immune organoids support preclinical evaluation of cancer immunotherapies through comparative modelling and translational investigation

**DOI:** 10.3389/fonc.2026.1988538

**Published:** 2026-10-29

**Authors:** Yang Zhang, Zhuo Wang, Yuting Xiu, Hongyu Wang, Kangkang Zhao, Zhishen Chen

**Affiliations:** 1 Department of Oncology, Jilin Cancer Hospital, Changchun, China; 2 Department of Radiotherapy, Jilin Cancer Hospital, Changchun, China

**Keywords:** immune checkpoint inhibitors, immunotherapy, precision oncology, preclinical models, tumor immune organoids, tumor microenvironment

## Abstract

The response of cancer patients to immunotherapy varies significantly. However, there is still a lack of functional models that can accurately predict efficacy *in vitro* while preserving individual tumor immune characteristics of patients. This to some extent restricts the development and clinical application of precision tumor immunotherapy. Patient-derived tumor organoids, as a three-dimensional preclinical model, can compensate for the shortcomings of traditional two-dimensional culture and animal models in tissue structure and microenvironment simulation, and better preserve the spatial relationships and interactions between tumor cells, immune cells, matrix components, and extracellular matrix. Therefore, it has gradually become an important tool for immunotherapy research. This article reviews four types of models: endogenous immune-intact tumor organoids, immune cell-reconstituted co-culture models, vascularized stromal tumor immune organoids, and microfluidic organoid-on-a-chip platforms. The focus is on comparing their detection indicators, model fidelity, and experimental reproducibility, and discussing their applications in predicting immunotherapy efficacy and studying tumor immune resistance mechanisms. At present, such models still have some shortcomings, including a gradual decrease in immune cells during *in vitro* culture, insufficient reconstruction of vascular and stromal structures, lack of unified experimental procedures, limited scale preparation, and insufficient evidence for prospective clinical validation. In response to the above issues, the introduction of artificial intelligence, multi-omics analysis, and gene editing technology may provide new research ideas for the optimization and clinical translation of tumor immune organoids, and provide reference for precision tumor immunotherapy.

## Graphical Abstract

**Figure:**
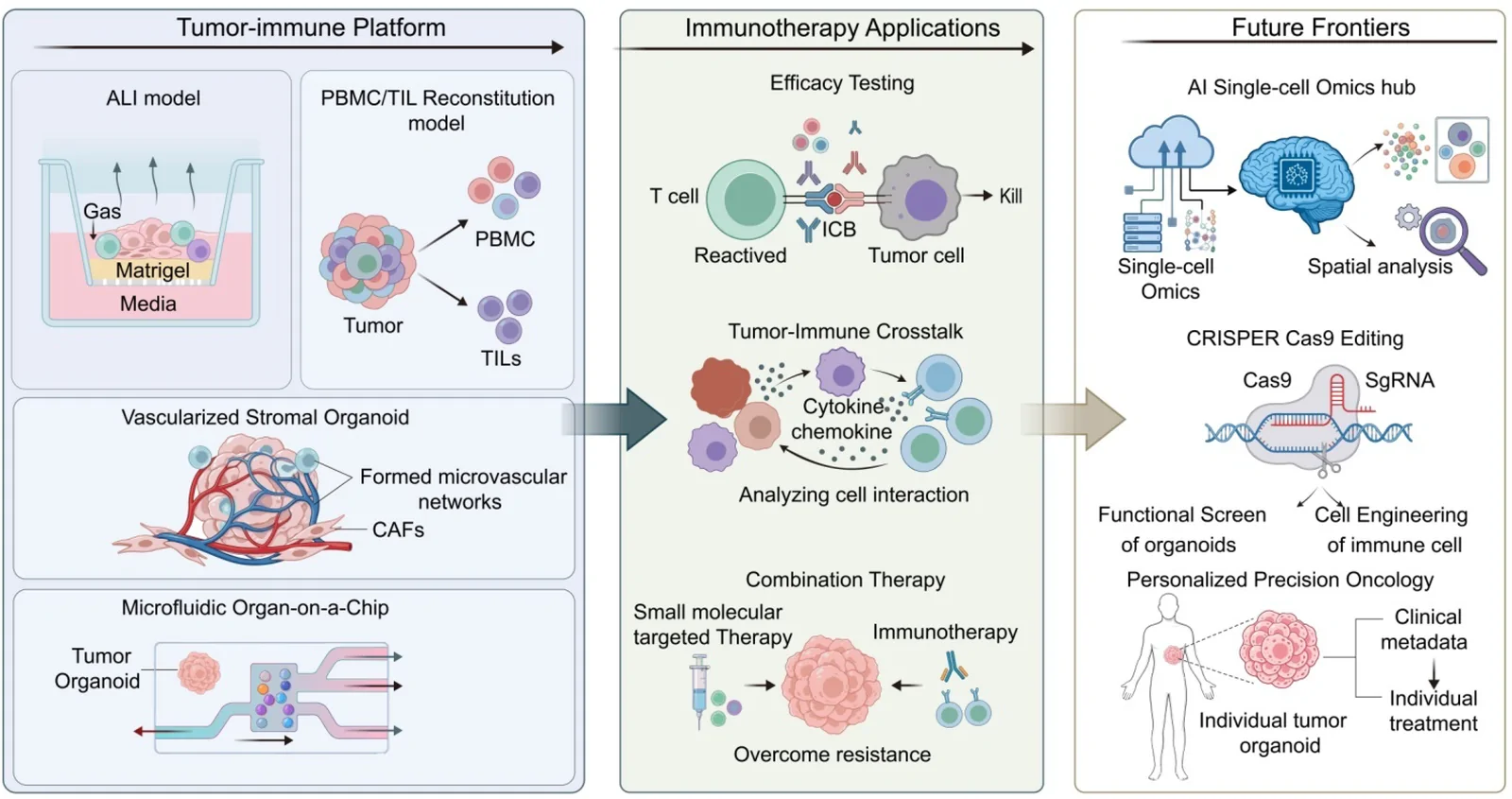

## 1 Introduction

In recent years, tumor immunotherapy has made continuous progress, but the proportion of patients who can truly benefit in the long term is still not high. Even if some patients show good response in the early stages of treatment, drug resistance may still occur in the future. The reason may be related to the inherent resistance mechanism that already exists before treatment, or it may be the acquired resistance gradually formed during the continuous treatment process (1, 2). At the same time, tumors exhibit significant differences within the same patient and between different patients, and individual immune responses are also not consistent. These factors collectively increase the uncertainty of immunotherapy efficacy and pose certain difficulties for clinical application (3–5).

The limitations of existing preclinical research methods are one of the important reasons for the high failure rate of immunotherapy drugs in clinical trials (6). The commonly used two-dimensional cell lines and patient derived xenograft (PDX) models can be used for partial mechanism and efficacy studies, but they are difficult to simultaneously restore the pathological and physiological states of the human immune system, tumor tissue, as well as the complex composition and heterogeneity of the tumor microenvironment (TME) (6). Therefore, there is often a deviation between the results obtained from these models and the actual efficacy and toxicity reactions of patients (6). In addition, genomic instability and differences within and between tumors can continuously alter TME, thereby participating in drug resistance formation, disease progression, and treatment failure (7). Based on these issues, preclinical research needs to establish experimental models that better reflect individual patient differences while preserving their tumor microenvironment characteristics, in order to improve the reliability of drug screening, safety evaluation, and immune therapy response prediction, and provide more effective experimental evidence for anti-tumor drug development.

Organoids are a type of three-dimensional (3D) multicellular structure formed *in vitro*, derived from pluripotent stem cells, as well as tissue resident stem cells and progenitor cells isolated from normal or diseased tissues. Through self-organizing growth, organoids can retain some key cellular components and to some extent present the structural characteristics and biological functions of the original tissue or organ. With the continuous development of organoid culture technology, relevant biological sample libraries are gradually being used for disease progression research and precision medicine exploration. At present, organoid models have been applied in research processes such as disease simulation, treatment target screening, preclinical drug testing, efficacy assessment, and safety evaluation.

As the limitations of traditional preclinical models in tumor immune research gradually become apparent, tumor immune organoids are beginning to receive attention. Unlike conventional epithelial organoids that predominantly recapitulate neoplastic epithelial compartments while lacking a functional immune compartment, tumor immune organoids are three-dimensional culture systems that integrate autologous immune populations and stromal components. This enables them to preserve genetic information, cellular heterogeneity, and microenvironment components from patient tumor tissue, thereby recapitulating the TME as well as reciprocal tumor-immune crosstalk, immune evasion, treatment response and acquired resistance. By providing experimental conditions closer to the actual human physiological situation, tumor immune organoids are essential for improving the reliability of immunotherapy response prediction and individualized treatment assessment (8). In this field, this article first summarizes the evolution process and main construction forms of tumor immune organoids, and then summarizes their applications in evaluating the effectiveness of immunotherapy. The article also analyzes the problems that still exist in the current model, such as the lack of immune components, inconsistent experimental procedures, difficulty in maintaining long-term stability of immune cells, and insufficient batch preparation capabilities. Finally, combining the development of artificial intelligence, multi-omics, gene editing, and personalized therapy, the study discusses the possible research directions for tumor immune organoids in the future, providing reference for the further improvement of related models and their clinical applications.

## 2 Model architectures of tumor organoid-based immune platforms

Traditional tumor organoids are usually dominated by malignant epithelial cells, with insufficient preservation of immune cells and matrix components (9). Due to the lack of these key components, the model is difficult to accurately depict the infiltration process of immune cells into tumor tissue, and cannot fully reflect the interactions between different cells in the tumor area and the actual response of patients to immunotherapy (9). Especially the human tumor microenvironment exhibits significant heterogeneity and constantly changes with disease progression, basic organoids compose of a single cellular component are difficult to replicate the true cellular composition and immune status of a patient’s tumor.

To address the aforementioned shortcomings, tumor organoid models have gradually evolved from simple patient derived organoids to immune functional culture systems that simultaneously introduce immune cells, stromal cells, extracellular matrix, and vascular related components (10). This type of model can better preserve the spatial structure and some biological characteristics of the primary tumor, providing experimental conditions closer to physiological states for observing T cell activation, immune regulation, and interactions between multiple cells (10). Due to its higher degree of simulation of the immune microenvironment in the body, tumor immune organoids have been used to study the relationship between tumors and the immune system, evaluate the efficacy of immunotherapy, and provide support for precision oncology research.

### 2.1 Endogenous immunepreserving tumor organoid cultures

#### 2.1.1 Air-liquid interface tumor immune organoids

Conventional tumor organoid cultures consist exclusively of neoplastic epithelium, devoid of intratumoral immune and stromal subsets, and thus fail to recapitulate the intact tumor immune microenvironment and *in situ* tumor-immune crosstalk. By contrast, ALI culture preserves endogenous tumor-infiltrating immune cells and stromal fibroblasts. Neal et al. pioneered 3D ALI culture across multiple cancer types, generating patient-derived organoids (PDOs) that stably retain native immune populations including T cells, B cells, NK cells and macrophages (10). Built on a double-layer collagen gel Transwell system with the apical gel surface exposed to air and the basal side immersed in medium ALI PDOs faithfully maintain the original T-cell receptor repertoire, clonal diversity and expression profiles of tumor-infiltrating lymphocytes (10). Critically, these models replicate clinical checkpoint blockade: anti-PD-1 (programmed death-1)/PD-L1 expands tumor-specific T cells and induces cytotoxicity (10). In colorectal cancer, ALI organoids preserve native 3D architecture, immune infiltration, and mutational landscapes (11). Nivolumab induced Treg expansion-a resistance hallmark absent in co-cultures confirms that intact tumor immune interactions are essential for evaluating immunotherapy and dissecting resistance (11).

Nevertheless, ALI organoids have inherent limitations. First, the protocol is technically demanding with low inter-laboratory reproducibility and no unified standardization (12). Second, long-term culture causes progressive viability loss and phenotypic drift in endogenous immune and stromal cells (13). Continuous exposure to persistent antigen stimulation, limited nutrient and oxygen diffusion, and the absence of systemic immune-cell replenishment may promote T cell dysfunction or exhaustion, characterized by reduced cytotoxic, cytokine secretive and proliferative capacity, and increased expression of inhibitor receptors such as PD-1, Tim3 and LAG3 (14). Progressive apoptosis of T cells and other immune subsets may reduce immune-cell abundance and alter the cellular composition of the model. The duration of reliable immunotherapy testing in ALI organoids is likely to be concentrated in the early culture phase, although the precise time window depends on the tumor type, donor characteristics, culture conditions, and experimental endpoint (10). Strategies to mitigate immune-cell decline may include supplementation with homeostatic cytokines such as IL2, IL-7 or IL-15, optimization of oxygen tension, and adjustment of extracellular-matrix composition (15, 16). However, the effects of these interventions on immune-cell survival and immunotherapy-response measurements in ALI organoids require further experimental validation. Third, the model lacks vasculature, fluid shear stress and systemic immune circulation, yielding incomplete tumor microenvironment recapitulation (17). Finally, the culture format is incompatible with high-throughput screening, and elevated costs further constrain large-scale applications (**Figure 1**) (18).

**Figure 1:**
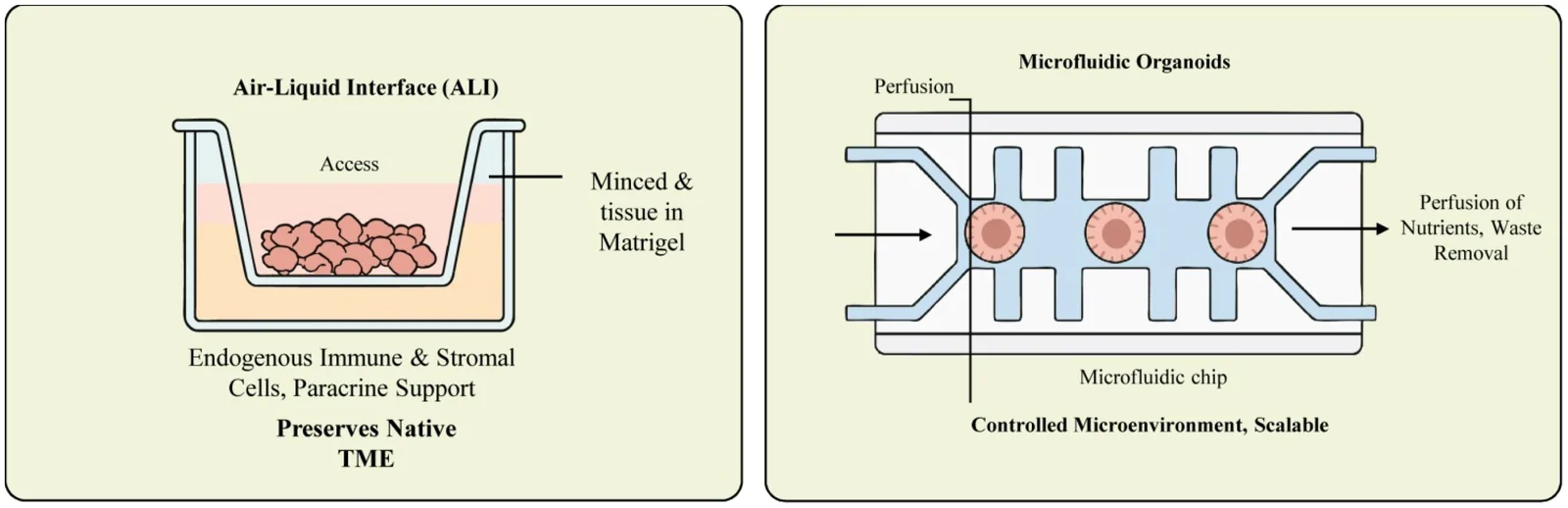
Schematic comparison of air-liquid interface and microfluidic organoid culture systems. ALI culture preserves native tissue architecture, stromal and immune components, and paracrine signaling by maintaining minced tissue in Matrigel at an air-liquid interface. Microfluidic organoid systems use controlled perfusion to deliver nutrients and remove waste, enabling scalable culture under a regulated microenvironment. Created with Adobe Illustrator.

#### 2.1.2 Microfluidic tumor immune organoids

In the traditional Matrigel embedding culture system, the supply of nutrients and the elimination of metabolic waste inside organoids mainly rely on passive diffusion. As the volume of organoids increases, this transmission method gradually becomes difficult to meet the needs of internal areas, especially leading to insufficient oxygen and nutrient supply in the central area (19). Microfluidic platforms provide a new solution to this problem by establishing a perfusion environment similar to physiological cycles (19). A continuously flowing culture medium can continuously replenish oxygen and nutrients, while taking away metabolic products, thereby providing organoids with more stable and *in vivo* growth conditions (20). Although dynamic perfusion can improve nutrient and oxygen delivery, flow-induced mechanical forces may influence the viability, migration, adhesion, and activation state of immune cells in microfluidic tumor-immune organoid systems (21–23). Excessive or rapidly fluctuating shear stress may compromise immune-cell survival or alter immune-cell phenotype, although the magnitude and direction of these effects depend on the immune-cell subtype, exposure duration, local flow field, and organoid or matrix architecture (21, 24). Therefore, flow conditions should be optimized through a combination of computational fluid-dynamics modeling and experimental calibration (21, 22). Models should incorporate channel geometry, fluid viscosity, matrix permeability, and, where appropriate, porous-media transport to estimate both channel wall shear stress and interstitial flow within the extracellular matrix (21, 24). Pressure-driven pumps may reduce the periodic flow fluctuations associated with peristaltic pumps, but pressure transients and air bubbles must still be controlled through appropriate tubing, flow resistance, and bubble-trap designs. Importantly, flow rate values cannot be directly generalized across platforms because the same flow rate may produce substantially different shear stresses in chips with different geometries. Reported values such as 0.1-1μL/min or 5-10μL/min should therefore be regarded as device-specific examples rather than universal operating ranges. For systems incorporating fragile immune populations, including tumor-infiltrating lymphocytes (TIL), relatively low and stable flow rates may be initially tested, followed by stepwise adjustment based on cell viability, migration, cytokine secretion, and functional-marker expression (25–27). Real-time or time-resolved assessment of these parameters is necessary to confirm that the selected perfusion conditions preserve immune-cell phenotype and function (25). The core of microfluidic technology is to use microfabrication techniques to precisely control microscale fluids. In actual cultivation, a small number of tumor cells can be densely seeded into chips with microporous structures, achieving miniaturization and integration of the organoid culture process (28). This method helps to improve the stability of organoid formation and the controllability of the experimental process, making it more suitable for high throughput screening and detection (29). At present, the commonly used materials for microfluidic chips include polydimethylsiloxane (PDMS), polymethyl methacrylate (PMMA) and hydrogel. Among them, PDMS has become a commonly used chip preparation material due to its good breathability, biocompatibility, and processing convenience (30).

Diverse microfluidic organoid platforms have been developed, demonstrating significant advantages in mimicking *in vivo* microenvironments and conducting drug screening. Li et al. developed a chip-free droplet microfluidic platform termed OsciSphere, which achieves deterministic and uniform bioassembly of 3D multicellular systems based on Weber number-induced inertial forces (20). The patient-derived organoids fabricated by this platform allow efficient infiltration of autologous immune cells and support quantitative evaluation of PD-1 blockade immunotherapy (20). Sun et al. used microfluidic devices to test the antitumor efficacy of recombinant oncolytic adenovirus AD4-GHPE in patient derived pharyngeal and breast cancer organoid models, and conducted a series of functional validations (31). Choi et al. established a microfluidic organoid platform for scarce PDAC biopsy samples, whose derived organoids match gold standard Matrigel-cultured counterparts in genotype and phenotype while featuring uniform spheroids, low cell demand and Matrigel independence, and this system enables reliable screening of chemotherapeutics and evaluation of NK cell-based combination immunotherapy (32).

While tumor organoid microfluidic platforms have significant advantages in modeling physiological niche, achieving dynamic perfusion, and improving the predictive accuracy of preclinical drug screening, significant barriers remain that impede their translation from engineering laboratories to routine clinical and research settings (20, 33). Traditional microfluidic systems are poorly compatible with viscous, temperature-sensitive matrices such as Matrigel, which can clog microchannels and cause uneven matrix distribution, compromising organoid viability, differentiation, and experimental reproducibility (34). Furthermore, the operational complexity of dynamic perfusion systems presents substantial challenges for routine laboratory use. Maintaining stable flow requires precise control of pumps, tubing, and flow rates, demanding specialized expertise and constant monitoring. Flow inconsistencies, bubble formation, and system failures can compromise experimental outcomes and reduce throughput. These technical demands make microfluidic platforms far less user-friendly than conventional static culture methods, creating significant adoption barriers for laboratories without dedicated microfluidics expertise. Economic barriers are equally substantial. Platforms like OsciSphere and other droplet microfluidic systems rely on expensive customized equipment, specialized hardware, and proprietary components not readily available through standard laboratory suppliers (20, 33). The initial capital investment can be prohibitive for many academic and clinical laboratories, while ongoing costs for maintenance, consumables, and trained personnel further limit accessibility. These financial constraints significantly hinder the democratization of microfluidic technology and its integration into standardized workflows (33, 35). Although microcavity arrays meet the requirements of standard culture procedures, their scalability is insufficient and they cannot prepare uniform cell spheres (20, 33). In addition, common technical issues such as droplet coalescence, low sample transfer efficiency, and operational complexity further limit the practicality of the system (36). These defects not only lead to high operating costs and poor experimental reproducibility, but also seriously hinder the widespread application of traditional microfluidic platforms in translational cancer research (**Figure 1**).

### 2.2 Immune cell-reconstituted tumor organoid co-cultures

#### 2.2.1 Tumor organoid-PBMC co-culture systems

T cells, pivotal for cancer immunotherapy, are constrained by current techniques in personalized expansion and tumor crosstalk studies. To faithfully recapitulate TME dynamics *ex vivo*, researchers have developed organoid-PBMC (peripheral blood mononuclear cells) co-culture models (37, 38). This practical and expandable platform is now widely applied to explore crosstalk between tumor cells and immune cells, as well as assess the efficacy of immunotherapy.

Three typical co-culture systems have been established. The fully embedded Matrigel culture model can promote T cell and NK cell infiltration to clear tumor cells, making it very suitable for studying immune activation, tumor immune escape, and dynamic interactions between tumors and the immune system (39, 40). This partitioned culture system physically isolates tumor organoids from PBMC, facilitating the analysis of indirect immune responses regulated by cytokines and chemokines (41). The direct co-culture method without Matrigel can rapidly generate tumor specific effector T cells by eliminating physical barriers through customized culture media, effectively detecting T cell activation status, cytokine profile, and anti-tumor cytotoxicity (42, 43). The first tumor organoid peripheral blood lymphocyte co-culture system has emerged, which can efficiently expand tumor reactive T cells from peripheral circulation and evaluate their tumor antigen reactivity and killing ability (43). Based on this method framework, subsequent research further improved the technical details and established a standardized operating process for the co-culture model. The co-culture system consists of two core stages (44): firstly, in the presence of IFN-γ- stimulated autologous tumor cells, tumor reactive T cells in peripheral blood are expanded through an initial co-culture for two weeks (44). The collected T cells will then undergo functional evaluations triggered by tumor recognition, including IFN-γ secretion and degranulation reactions, as well as their killing ability against tumor organoids (44). In non-small cell lung cancer (NSCLC) and microsatellite unstable colorectal cancer (CRC) samples, the system can successfully cultivate tumor responsive CD8^+^T cell populations within two weeks, with a success rate of approximately 33% to 50%. However, this limited success rate is influenced by multiple factors, including initial biopsy sample quality, disease stage, initial cell yield, tumor mutational burden, and the presence of pre-existing tumor-reactive T cell clones in peripheral blood. The modest success rate raises important questions about whether the current system is sufficiently robust to support widespread personalized medicine applications, particularly in settings requiring high-throughput screening or where patient samples are limited. Nonetheless, this co-culture system, providing a reliable *in vitro* functional evaluation platform for personalized T cell immunotherapy (44). Compared to traditional two dimensional cultivation methods, this co-culture system can better preserve the morphological structure and proliferation ability of tumor tissues, and can achieve real-time observation and quantitative analysis of individualized anti-tumor immune responses. This system provides a reliable tool for exploring tumor immune mechanisms, screening immunotherapy targets, and evaluating personalized immunotherapy, with significant clinical translational potential.

Co-culture of tumor organoids with PBMCs can to some extent reproduce the tumor immune microenvironment and assess immune therapy response. However, there are still many limitations to the widespread application of this system. Firstly, existing cultivation relies heavily on Matrigel. It is difficult to fully restore the complex composition and physical structure of the extracellular matrix in the original tumor tissue. This may alter the migration and infiltration behavior of immune cells, thereby weakening the model’s ability to reflect the *in vivo* state (45). Meanwhile, commercialized Matrigel is derived from mouse tumors and its protein and growth factor components are not fully understood, which may cause non-specific immune responses and interfere with experimental results (43). To address this limitation, several alternatives with defined compositions have been developed, including synthetic hydrogels based on polyethylene glycol (PEG) or poly (lactic-co-glycolic acid) (PLGA), purified silk fibroin matrices, recombinant collagen scaffolds, and alginate-based systems (46–48). These defined matrices offer improved reproducibility, reduced batch-to-batch variability, and minimized xenogeneic contamination, though optimization of mechanical properties and cell-matrix interactions remains necessary for each specific application. Secondly, a critical limitation lies in the inherent differences between circulating PBMCs and TILs in TCR diversity and cellular phenotype (37, 45). Autologous PBMCs often fail to generate sufficient tumor-reactive T cells, as tumor-specific clones are typically less frequent in peripheral blood than at the tumor site (49). Moreover, peripheral blood contains suppressive populations including MDSCs and Tregs, which can skew immune responses within the co-culture system (50, 51). In contrast, TILs represent T cells that have already undergone selection and expansion within the tumor microenvironment and thus exhibit enriched tumor reactivity, appropriate homing receptor expression, and functional adaptation to the TME, their isolation requires invasive procedures with often low yields, and expansion protocols are labor-intensive (52, 53). The PBMC-organoid co-culture approach thus represents a practical compromise, trading biological fidelity for accessibility and scalability (54). Future hybrid strategies, such as engineering peripheral T cells with tumor-specific TCRs derived from TIL repertoires, may help address these limitations (55, 56). In addition, existing organoids often lack perfusable vascular structures. As the volume of organoids increases, there is a lack of internal oxygen and nutrient supply, which can lead to central necrosis and increased oxidative stress, thereby affecting the original biological state of tumor cells (57). In response to these issues, previous studies have started using purified or artificially synthesized matrices with clear components, increasing the co culture of multiple cells, and combining microfluidic technology to construct a culture system with vascularization and perfusion conditions (32). However, the lack of unified operating procedures and evaluation criteria among models compromises repeatability and cross-study comparability. Establishing standardized protocols and a complete evaluation system is thus essential for improving model stability and promoting clinical translation (**Figure 2**).

**Figure 2:**
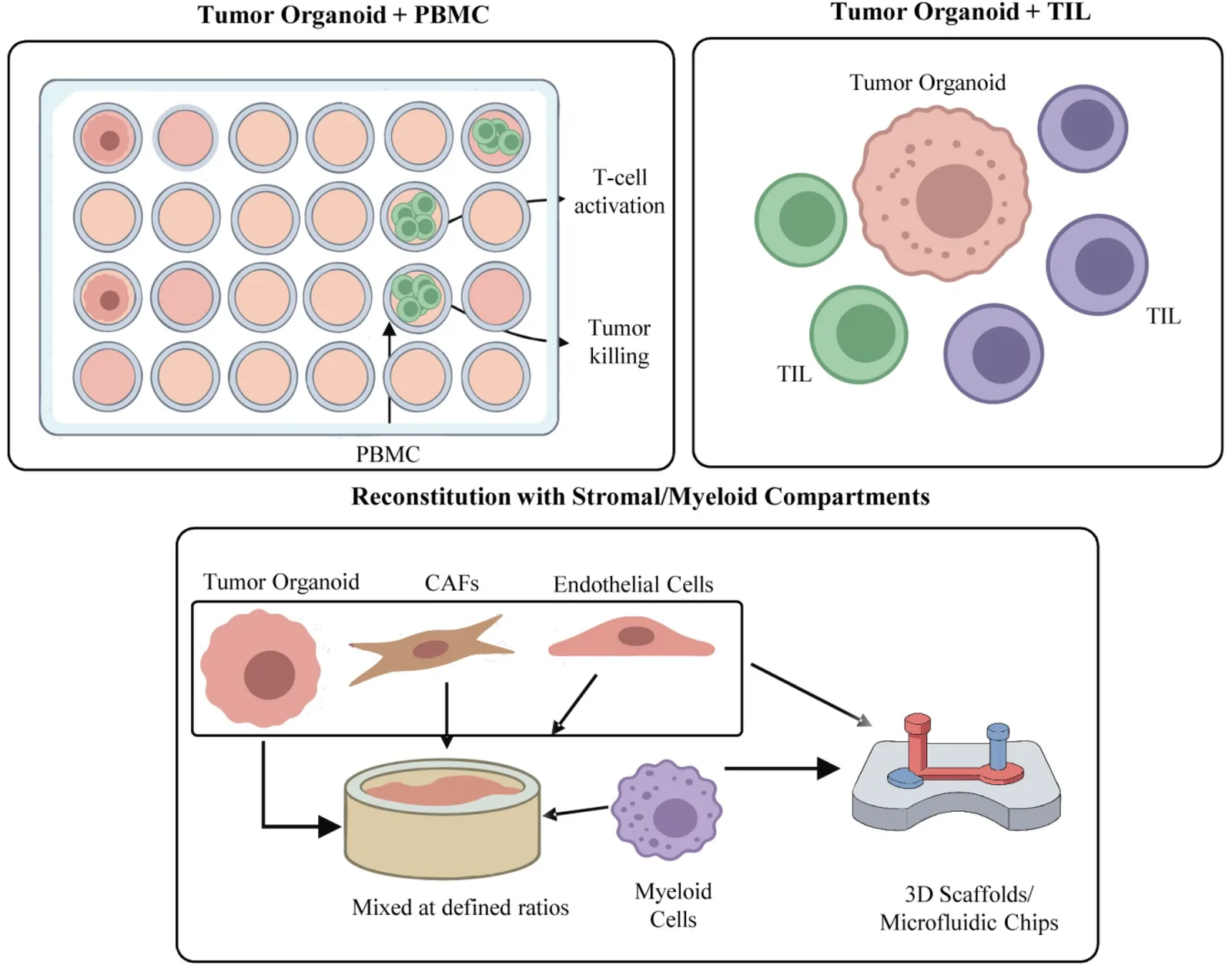
Schematic workflow of reconstituted organoid co-culture models. Organoids are first established in multi-well culture formats and then combined with selected tumor microenvironment components, including immune cells (PBMC or TIL), stromal cells, and other supporting cell populations. These reconstituted co-culture systems allow defined cell types to be introduced into organoid cultures to model tumor immune and tumor stromal interactions. Created with Adobe Illustrator.

#### 2.2.2 Tumor organoid-TIL co-culture systems

TILs represent a population of mononuclear cells naturally residing within the tumor microenvironment. Moreover, it serves as a pivotal biomarker for predicting responsiveness to cancer immunotherapy (58). TILs isolated from tumor lesions can be subjected to large scale *ex vivo* expansion and culture. After adoptive transfer of expanded cells back into subjects, the tumor-specific immune microenvironment *in vivo* can be effectively remodeled, thereby mediating potent tumor-specific cytotoxic killing effects.

To establish tumor immune evaluation models, surgically resected tumor specimens are split into two portions. One fraction is used for patient-derived tumor organoid culture, while the other is processed via mechanical trituration and enzymatic digestion to generate single-cell suspensions (59). CD45^+^ immune cells (primarily TILs) are isolated from digested tumor debris using Ficoll density gradient centrifugation or CD45-based magnetic-activated cell sorting (MACS) (59). Purified autologous TILs are co-cultured with matched organoids to build a personalized tumor-immune model (60). Direct co-culture the most established platform-enables physical contact between organoids and TILs, closely mimicking *in situ* interactions (60). Most direct co-cultures use suspension format with shared medium (60). Pre-cultivation in separate media is required: TILs in RPMI 1640, organoids in stemness-supporting medium. These media are incompatible-immune medium lacks stemness signals for organoids, while organoid medium induces stress and impairs immune cell viability/expansion. To address this technical challenge, several strategies have been employed in practice (61). Tumor organoids are generally established and expanded in stem-cell-supporting media before immune-cell addition. Depending on the tissue of origin, these media may contain factors such as EGF, Noggin, and R-spondin1 (10, 62, 63). For co-culture with TILs or other tumor-reactive T cells, culture conditions may be adjusted to support both organoid viability and lymphocyte function, for example by modifying the basal medium, omitting selected growth factors, or limiting the co-culture period (43, 44) However, medium composition and co-culture conditions vary among models, and no standardized hybrid medium has been established. Therefore, these parameters should be optimized empirically according to the experimental readout.

To investigate the interaction patterns between lymphocytes and tumor cells, Shin JH et al. established a co-culture system of colorectal cancer organoids and TILs. Their results verified that the endocytosis-mediated transfer of lymphocyte membrane proteins to tumor cells exhibits obvious time dependence, and actin polymerization as well as the phosphatidylinositol 3-kinase (PI3K) signaling pathway serve as indispensable regulatory components of this transport process (64). Emerging evidence suggests that tumor cells can acquire surface molecules from immune cells through trogocytosis or extracellular vesicle uptake, potentially enabling immune evasion by masking tumor-associated antigens or acquiring immune checkpoint ligands (65–67). Such molecular “camouflage” may allow cancer cells to evade cytotoxic T cell recognition or suppress immune activation (68, 69). However, the physiological significance of this process *in vivo* remains incompletely understood and warrants further investigation using more complex three-dimensional models and clinical validation studies to determine whether it constitutes a bona fide immune escape mechanism or is amplified under artificial culture conditions. Sui established organoid models using tumor specimens harvested from patients with microsatellite instability-high colorectal cancer, and performed co-culture assays with autologous tumor-infiltrating lymphocytes as well as T lymphocytes differentiated from PBMC, respectively (70). This study verified that patients developing local inflammatory responses after treatment had a higher risk of tumor progression. The underlying mechanism was linked to a neutrophil-induced immunosuppressive microenvironment, whose inhibitory effect blunted the therapeutic efficacy of immune checkpoint blockade agents. To obtain abundant tumor specific TILs, Li Zhilang’s team established an *ex vivo* co-culture platform and applied iterative stimulation with patient-derived autologous colorectal tumor organoids. Relevant experimental data demonstrated that this organoid-TIL co-culture platform can rapidly identify tumor-specific TCRs. Then, it can offer key technical support for the translational development of TCR-T immunotherapy (71). An individualized *in vitro* platform can also be constructed by co-culturing expanded autologous TILs with matched colorectal cancer organoids from the same patient to predict clinical benefits of neoadjuvant chemoradiotherapy (72).

Organoid-TIL co-culture models effectively recapitulate patient-specific tumor heterogeneity and tumor immune crosstalk, serving as robust *in vitro* research tools. However, these simplified systems fail to fully mimic the complexity of native TME. The *in vivo* TME comprises intricate paracrine signaling, endogenous extracellular matrix (ECM), microvascular networks, and cancer-associated fibroblasts (CAFs), which are critical drivers of tumor progression and are absent in conventional *in vitro* models. To overcome these limitations and improve model bionic fidelity, emerging research integrates native ECM derived from primary or metastatic tumor tissues into co-culture systems. Such biomimetic optimization that restores TME structural and functional authenticity has become a key frontier in preclinical tumor modeling and immunotherapy research (**Figure 2**).

#### 2.2.3 Stromal and vascularized tumor immune organoid models

Tumors develop within a complex immunosuppressive tumor microenvironment composed of key immune cells, abnormal vasculature, and ECM (73). These components drive tumor proliferation and immune-evasive niches, regulating tumor progression and immune escape (73)). Abnormal angiogenesis sustains tumor growth by delivering nutrients (73). CAFs, central to ECM remodeling, promote tumor cell proliferation and form a dense physical barrier (74) that impedes effector T cell infiltration and impairs host anti-tumor immunity (74). Avascularity remains a critical bottleneck in organoid culture, causing nutrient deprivation, hypoxia, and waste accumulation that trigger premature apoptosis and differentiation (75, 76). The absence of functional vasculature limits physiological immune-cell extravasation, endothelial regulation of leukocyte adhesion through molecules such as ICAM-1 and VCAM-1, and perfusion-dependent drug delivery, thereby restricting the ability of organoid models to recapitulate immunotherapy responses (77). Current vascularization strategies include co-culture with endothelial cells, supplementation with proangiogenic factors, and microfluidic or perfusion-based systems (78–80). However, the resulting vascular networks often show incomplete lumen formation, limited pericyte coverage, insufficient long-term stability, and inadequate reproduction of the irregular and highly permeable architecture characteristic of tumor vessels (78–81). iPSC-derived vascular cells may improve experimental reproducibility and enable more controlled vascular engineering, but their stable integration with tumor organoids and immune-cell populations remains technically challenging (79, 80). Functional lymphatic components are also rarely incorporated into these platforms, limiting the modeling of antigen drainage and immune-cell trafficking to lymph nodes (82). Thus, vascularized organoid systems have considerable potential, but further work is needed to establish stable, perfusable, and immunologically functional vascular and lymphatic networks.

Apoptosis and premature differentiation limit the success of organoid culture-a problem rooted in avascularity, which restricts nutrient/oxygen supply and waste removal, impairing growth (75, 76). Adding pro-angiogenic elements extends culture time, supports organoid maturation, and improves structural complexity and physiological relevance.

The bottom-up reconstruction method is the main strategy for simulating the TME *in vitro* using standardized hierarchical processes. First, the patient tumor organoids derived from pure epithelium were constructed using matrix gel and other extracellular matrix hydrogels. Simultaneously isolate and amplify stromal components including cancer-related fibroblasts and endothelial cells independently. The primary macrophages used to construct the medullary compartment are obtained by differentiation from autologous PBMC, or by mechanical dissociation and fluorescence activated cell sorting techniques from tumor tissue. After completing the preparation and amplification of all cell components, tumor organoids, stromal cells, and myeloid cells are mixed in the optimal ratio and then re-embedded in three-dimensional scaffolds or cultured on microfluidic chips and ultra-low adhesion plates to construct TME models. This multi-lineage assembled *in vitro* model can achieve controllable and accurate TME reproduction and intercellular communication simulation, providing a reliable platform for evaluating the therapeutic effect of multispecific antibodies. At the same time, DeNardo and colleagues demonstrated that a multicellular pancreatic cancer co-culture model to verify that targeted elimination of M2 polarized macrophages through CSF1R inhibitors can effectively reverse TME mediated chemotherapy resistance (83). Zhang Feng et al. developed a gravity perfusion immune infiltrating tumor spheroid model that used matrix embedding of fibroblasts and pericytes to enhance vascular barrier function (84). Within a week, this process can induce time-dependent and tissue-specific recruitment of monocytes, transendothelial differentiation, and formation of cancer macrophage heterospheres in breast and liver tumor spheroids (84).

However, there are still significant technical limitations to the multi-component co-cultured tumor model. Firstly, myeloid cells such as macrophages have a shorter *in vitro* culture cycle and are prone to phenotype drift. Macrophages exhibit exceptional plasticity and can rapidly switch between polarization states, such as transitioning from pro-inflammatory M1 to immunosuppressive M2 phenotypes or vice versa, in response to microenvironmental cues (85–87). If there is a lack of endogenous cytokine network support, its gene expression profile and tumor specific functional characteristics will rapidly deteriorate after short-term cultivation (86, 87). Although optimized organoid co-culture conditions and supplementation with factors such as M-CSF, IL-4, IL-10, or tumor-conditioned medium may help preserve macrophage-associated phenotypes, the stability of markers such as CD206, CD163, and arginase-1 remains highly dependent on the tissue source, culture medium, matrix composition, and experimental duration (85–88). Therefore, functional analyses should be conducted within the validated polarization window. Secondly, it is difficult to standardize the cell ratio in co-culture systems, and artificially set cell ratios cannot accurately simulate the physiological spatial distribution and heterogeneity of the tumor microenvironment *in vivo* (9, 16, 89). In addition, traditional static culture systems lack vascular perfusion and metabolic clearance functions, leading to local accumulation of lactate, ATP depletion, and accumulation of other metabolic waste, thereby causing non-specific cytotoxicity (90–92). Furthermore, tumor cells, stromal cells, and myeloid cells have different or even contradictory demands for growth factors, and a single culture medium cannot support the survival of all cell types, affecting overall cell viability (9, 93). To address this fundamental incompatibility, several strategies have been explored (94, 95)). One approach uses staged medium exchange: cells are first expanded separately in optimized media, then combined in a compromise “co-culture medium” with reduced but balanced growth factor concentrations (e.g., minimal M-CSF for macrophages, reduced R-spondin/Noggin for organoids, and endothelial supplements) (96, 97). Alternatively, microfluidic platforms enable spatially segregated perfusion, delivering cell-specific media to distinct compartments while preserving paracrine signaling (98, 99). Gradient-based systems-such as hydrogels with spatially controlled growth factor release or organ-on-chip devices-show promise for maintaining multi-lineage viability (100, 101). Conditioned medium strategies can partially recapitulate cellular cross-talk without direct medium conflict (102). However, no single solution fully replicates the dynamic, heterogeneous biochemical milieu of the native TME; the choice depends on experimental endpoints, culture duration, and throughput needs. In summary, these deficiencies limit the stability and experimental throughput of the model, and restrict its large-scale application in high-throughput drug screening (**Figure 2**).

#### 2.2.4 3D bioprinted tumor immune organoid models

As an innovative and advanced microfluidic control technology, 3D bioprinting of tumor models overcomes the inherent technical limitations of traditional PDMS based microfluidic systems (103, 104). This emerging biotechnology can highly restore the microstructure and pathological characteristics of *in situ* tumors, and its biomimetic performance is far superior to traditional *in vitro* experimental platforms (105). This bioprinting system can accurately reproduce tumor heterogeneity, physiological nutrient concentration gradients, and hypoxic microenvironment conditions (105). It can also construct core tumor structures including multicellular spheroids and necrotic areas (105). These precise biomimetic models provide a reliable experimental platform for studying how the spatial configuration of tumors affects drug responsiveness, invasion and metastasis ability, and overall treatment efficacy (105). Traditional microfluidic chips are difficult to accurately reproduce the complex composition and spatial distribution of ECM. 3D bioprinting technology can achieve targeted deposition of various bioactive matrix materials such as collagen, fibronectin, hyaluronic acid, and matrix glue, maximizing the restoration of the tumor physiological microenvironment and ensuring the authenticity and stability of *in vitro* tumor models.

In addition, thanks to its precise spatial cell arrangement ability, 3D bioprinting technology can achieve local construction of tumor hypoxic areas, effectively solving the long-standing shortcomings of traditional microfluidic devices in simulating oxygen gradients (106). This innovative modeling strategy provides strong support for the systematic study of the impact of hypoxic microenvironment on tumor malignant phenotype and sensitivity to chemotherapy and targeted therapy. At the same time, this biomanufacturing technology can efficiently achieve co-culture and spatial organization of immune cells and vascular endothelial cells, accurately reproducing key biological processes including immune cell infiltration, tumor immune cell interactions, and functional microvascular network formation (107–109).

However, 3D bioprinting still faces important technical challenges. Limited printing resolution may hinder the reconstruction of fine tumor microenvironmental architecture at the cellular and subcellular levels (110). In addition, shear stress generated during bioink extrusion can damage cells, induce cell death, or cause unintended immune-cell activation, thereby reducing model fidelity and confounding experimental results (111–113). T cells, NK cells, and macrophages may be particularly susceptible to shear-induced phenotypic changes that alter their functional states and interactions with tumor cells (114). Therefore, further optimization of bioink rheology, nozzle geometry, and printing parameters is needed to minimize shear stress while preserving printing fidelity (115).

Owing to these prominent advantages and ongoing efforts to address technical limitations, bioprinted tumor platforms serve as high-precision biomimetic systems for elucidating the molecular mechanisms underlying tumor immune escape, evaluating the therapeutic efficacy of immune checkpoint inhibitors, and clarifying the regulatory roles of angiogenesis in drug delivery and tumor metastasis progression (**Figure 2**; **Table 1**).

**Table 1:** Major types and characteristics of tumor immune organoid models.

Model type	Main components	Advantages	Limitations	Main research applications
Air-liquid interface (ALI) tumor immune organoids	Air-liquid interface (ALI) tumor immune organoids with their native immune microenvironment	Better preserves cellular composition and spatial architecture	Shortculture duration; limited standardization	PD-1/PD-L1 blockade; short-term drug response assessment (10, 11)
Tumor organoid + PBMC co-culture model	Patient derived tumor organoids co-cultured with PBMCs, T cells, or NK cells	Immune cells are readily accessible; suitable for high-throughput experiments	Limited site-specific matching between immune cells and the original tumor tissue	Immune cell-mediated cytotoxicity; evaluation of immune checkpoint inhibitors (41–43)
Tumor organoid-TIL co-culture model	Tumor organoids co-cultured with TILs	Preserves relatively strong tumor-specific immune responses	TIL abundance and activity are highly dependent on sample quality	Tumor antigen recognition; investigation of T-cell function (64, 70, 71)
Tumor organoid-myeloid cell co-culture model	Tumor organoids co-cultured with macrophages and other myeloid cells	Enables investigation of the immunosuppressive tumor microenvironment	Cell proportions and macrophage polarization states are difficult to control	Tumor-associated macrophages; mechanisms of immunosuppression (83, 84)
Vascularized or microfluidic organoid model	Tumor organoids integrated with vascular networks and/or fluidic circulation systems	Simulates immune cell recruitment, migration, and infiltration	Technically complex and relatively expensive	Immune cell transport; dynamic and time-dependent immune infiltration (20, 31, 32)
Bioprinted organoid array	Patient-derived tumor organoids with precisely controlled spatial organization	Facilitates standardization and parallel testing under multiple conditions	Requires specialized equipment and technical expertise	Personalized treatment selection; high-throughput drug screening (105)

## 3 Applications of organoids in translational research of tumor immunotherapy

### 3.1 Profiling T cell function and immunotherapy efficacy of ICIs

The development of immune checkpoint inhibitors (ICIs) was based on two important studies in the 1990s. Allison discovered the immunosuppressive effect of CTLA-4 (cytotoxic T lymphocyte associated antigen 4) (116), while Honjo confirmed that PD-1 is involved in immune negative regulation (117). Afterwards, ICIs were gradually used for the treatment of various malignant tumors and achieved certain results, but there are still a considerable number of patients in clinical practice who cannot benefit from them.

Currently, drugs targeting the PD-1/PD-L1 and CTLA-4/CD80/CD86 pathways have become important components of tumor immunotherapy (118). These drugs have shown good therapeutic effects in solid tumors such as melanoma (118, 119) and non-small cell lung cancer (120, 121) by relieving T cell suppression and restoring anti-tumor immune function. Representative drugs that have been approved include ipilimumab, nivolumab, pembrolizumab, and atezolizumab. With the continuous deepening of research on tumor immune mechanisms, new immune checkpoints such as TIGIT, TIM-3, LAG-3, VISTA, BTLA, and SIRPα have also been discovered, and monoclonal antibodies targeting these targets are currently in the clinical development stage (122, 123). These new targets provide new possibilities for expanding the scope of immunotherapy and overcoming drug resistance. Multiple preclinical studies have shown that relevant models can be used to evaluate individual patient responses under different immunotherapy regimens.

Currently, ICIs directed against the PD-1/PD-L1 and CTLA-4 axes serve as the cornerstone of clinical oncology (123, 124). Tumor organoids serve as ideal research platforms to investigate T cell bioactivity and therapeutic responses to immune checkpoint inhibitors. Multiple culture systems, including multi-organoid systems, patient derived tumor spheroids, ALI models and tumor stem cell cultures, have successfully reproduced the anti-tumor effects of anti-PD-1 agents under *ex vivo* conditions (10, 125–128). Utilizing an ALI culture methodology, Neal et al. propagated patient derived organoids (PDOs) that successfully retained endogenous immune populations. Their research findings indicate that anti-PD-1/PD-L1 blockade effectively reactivates tumor specific TILs to eradicate cancer cells, thus establishing the feasibility of using PDO to evaluate ICI efficacy and customize personalized treatment predictions (10). At the same time, Wan and colleagues evaluated monospecific and bispecific antibodies against PD-1/PD-L1 in high-grade serous cancer organoids (129). Their work not only emphasizes that natural killer (NK) cells are essential mediators in driving ICI mediated therapeutic performance, but also identifies BRD1 as a potential drug target (129). Overall, these milestones emphasize the practical utility of immune active tumor organoids in dissecting immunotherapy dynamics and accelerating drug discovery (**Figure 3**; **Table 2**).

**Figure 3:**
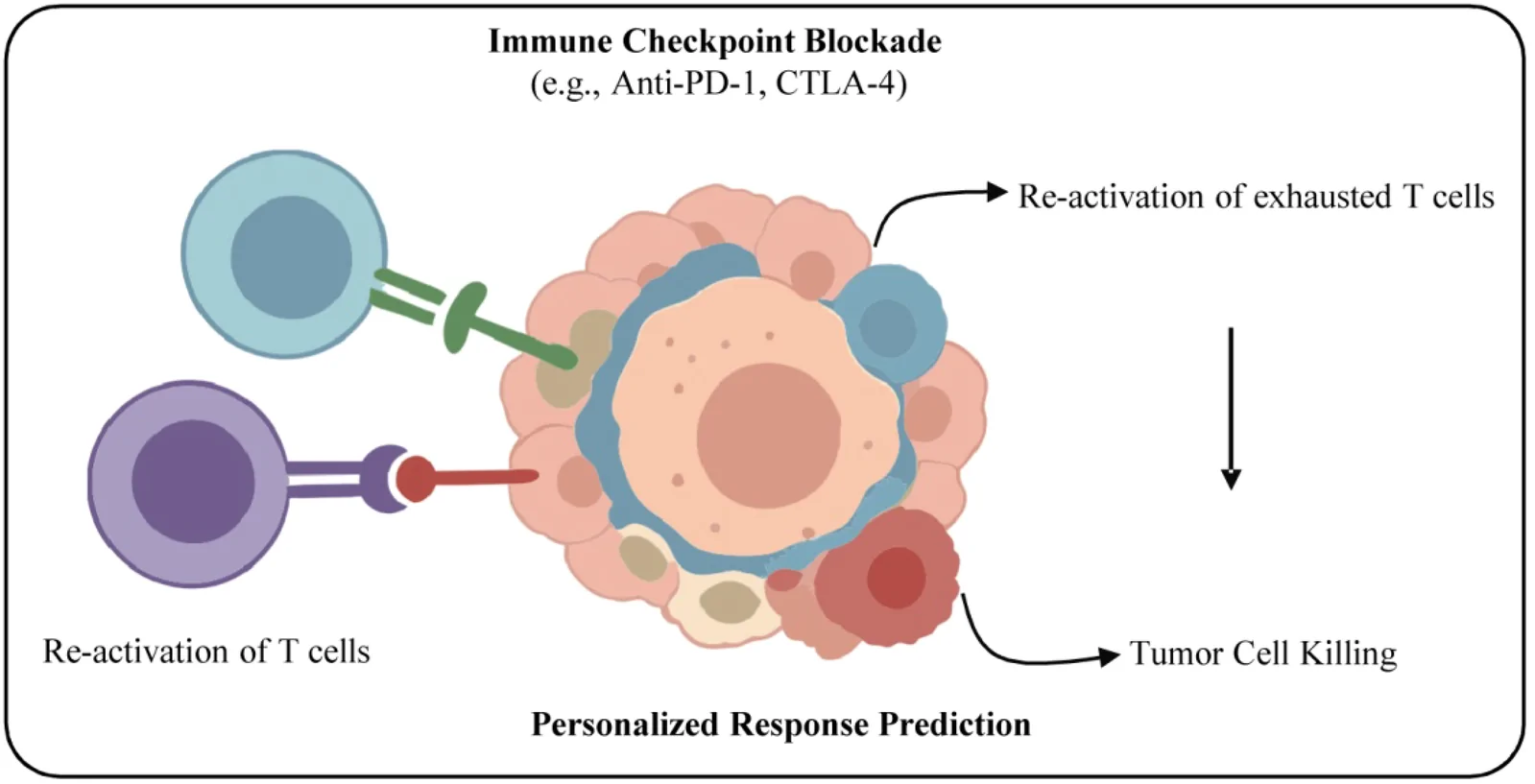
Tumor immune organoid model for evaluating T cell function and immune checkpoint blockade efficacy. Tumor organoids (pink/peach spheroid structure) co-cultured with T cells (purple cells, left) provide a functional platform to assess T cell activation, infiltration (blue cells surrounding and within the organoid), and cytotoxicity against tumor cells (red/brown cells indicating dying tumor cells). Exhausted T cells (light blue cells, top left) can be re-activated through immune checkpoint blockade (ICB) therapies (e.g., anti-PD-1, CTLA-4 antibodies, shown as green and purple Y-shaped symbols). This model enables preclinical testing of ICB therapies by monitoring treatment-induced enhancement of T cell-mediated tumor killing, thereby facilitating personalized response prediction. Created with Adobe Illustrator.

**Table 2:** Therapeutic strategies and core research questions in tumor immune organoid models.

Research strategy	Main research applications	Key quantitative assay	Standard evaluation timeframes
Profiling T-cell function and ICIs efficacy	Mechanisms of immune checkpoint inhibition and T cell functional restoration (10, 125–129)	Flow cytometry (CD8^+^, PD-1, IFN-γ, Granzyme B); ELISA (TNF-α, IFN-γ, IL-2); Live imaging (cytotoxic killing); Viability assays	24–72 h (cytokine release, initial activation); 5–7 days (cytotoxicity, proliferation); 14–21 days (sustained response, memory formation)
Bispecific antibodies/T-cell engagers	Efficacy and toxicity evaluation (132–136)	Flow cytometry (target depletion, CD25, CD69); ELISA (IL-6, cytokine storm markers); Live imaging (redirected killing); LDH release	6–48 h (immediate cytotoxicity, CRS onset); Efficacy assessment: 3–7 days (tumor reduction)
Combination therapy and resistance reversal	Resistance mechanisms and synergy assessment (137–139)	Flow cytometry (PD-1, TIM-3, Ki-67, Annexin V); Multiplex cytokine profiling; RNA-seq (resistance signatures); Spatial imaging	Baseline resistance: 0–3 days (pre-treatment profiling); Early response: 5–7 days (synergy assessment); Resistance reversal: 10–21 days (sustained sensitization)
Tumor-immune crosstalk mechanisms	Myeloid immunosuppression and TME remodeling (152, 154)	Flow cytometry (M1/M2, MDSCs, CAF markers); ELISA (IL-10, TGF-β, PGE2); Multiplex IF/IMC (spatial analysis); Metabolic assays	Early crosstalk: 24–72 h (initial cytokine secretion); Polarization dynamics: 5–7 days (M1/M2 shift, CAF activation); Chronic suppression: 14–21 days (established immunosuppressive TME)

### 3.2 Efficacy and toxicity assessment of multi-specific antibodies

Currently, the preclinical efficacy and toxicity evaluation of bispecific T-cell engagers (BiTEs) and trispecific antibodies mainly rely on conventional platforms such as tumor cell lines and rodent models, which have prominent limitations. Two-dimensional tumor cell lines lack 3D architecture, cellular heterogeneity and an intact tumor immune microenvironment, and thus fail to truly recapitulate the interactions between T cells and tumor cells (130). Consequently, they cannot accurately assess T cell infiltration, activation and bystander killing mediated by multispecific antibodies (130). In addition, discrepancies in antigen expression and immune pathways between rodents and humans often lead to underestimation of cytokine release syndrome (CRS) induced by BiTEs and trispecific antibodies (131). These animal models also cannot recapitulate the off-tumor toxicity of such antibodies against normal tissues including the intestines and lungs (131). The large deviations between results obtained from traditional models and clinical outcomes greatly hinder the screening, dose optimization and safety assessment of multi-specific antibodies (**Figure 4**; **Table 2**).

**Figure 4:**
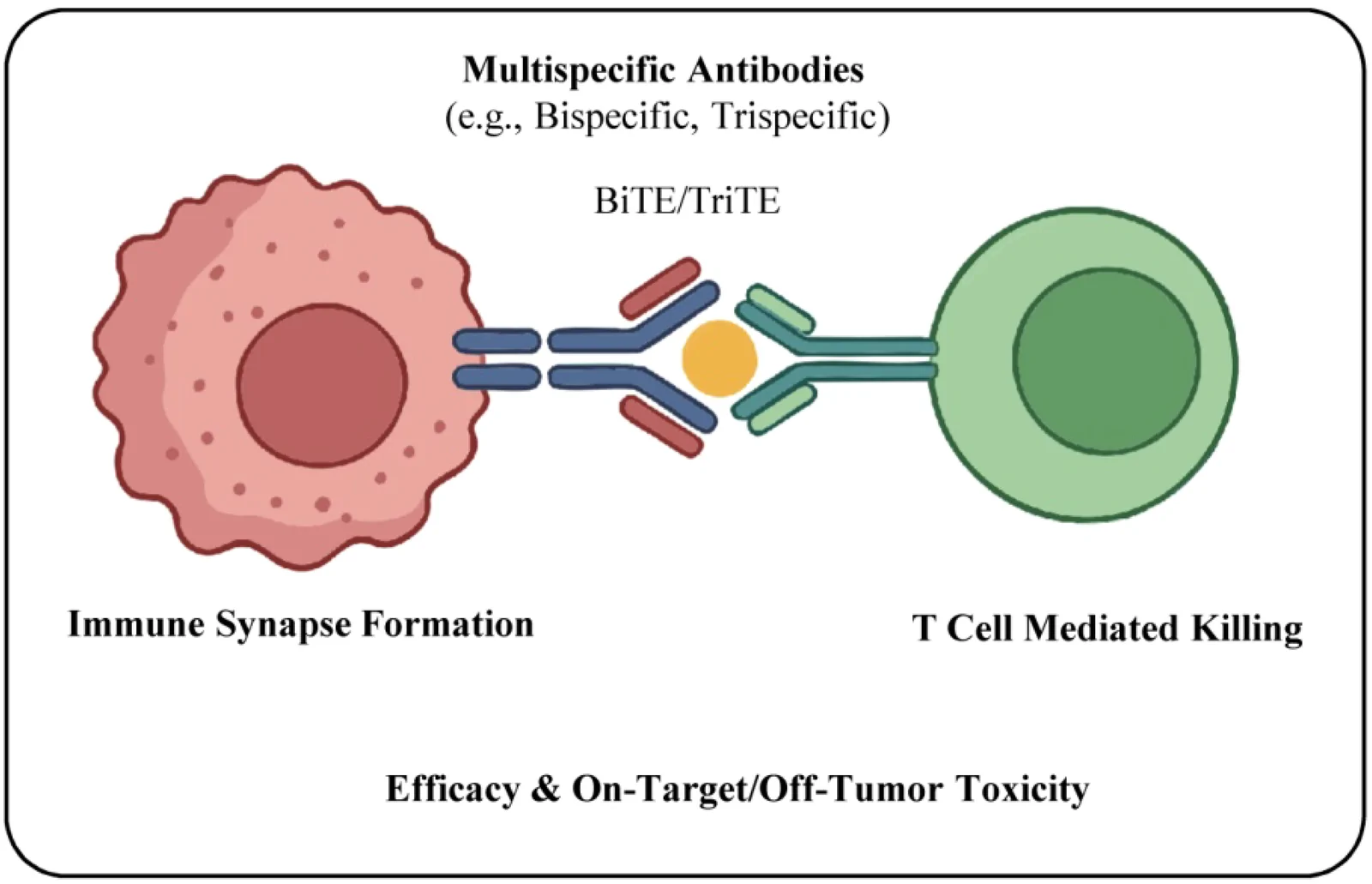
Evaluation of bispecific and trispecific T cell engagers using a tumor-immune organoid model. Tumor cells/organoids and T cells are co-cultured in the presence of bispecific or trispecific T cell-engaging antibodies, which bridge tumor-associated antigen(s) on cancer cells with T cell activating receptors. This interaction promotes T cell activation and tumor cell killing, enabling functional assessment of T cell engaging immunotherapies. Created with Adobe Illustrator.

PDOs co-cultured with autologous or allogeneic immune cells have overcome major bottlenecks of conventional evaluation models, emerging as an ideal platform for the preclinical research of multi-specific antibodies. These models retain the molecular signatures, spatial architecture and cellular heterogeneity of primary tumors, and reconstruct a human tumor immune microenvironment consisting of T cells and immunosuppressive cells. They can directly recapitulate the entire process of antibody-mediated T cell bridging and tumor cell killing (132–134). Compared with traditional models, this system enables quantitative evaluation of the antitumor efficacy of bispecific and trispecific antibodies, as well as simultaneous detection of inflammatory factors such as IFN-γ to predict the risk of CRS (133). When combined with matched normal tissue organoids, it can accurately identify off-tumor toxicity of candidate drugs (133). However, it is important to acknowledge that organoid models, being localized *in vitro* systems, have inherent limitations in predicting complex systemic phenomena. While they can detect cytokine secretion at the local tissue level, severe CRS (Grade 3+) is a multi-organ systemic inflammatory response involving dynamic cytokine amplification cascades, vascular endothelial dysfunction, and organ-organ interactions that cannot be fully recapitulated in isolated organoid cultures. Therefore, although tumor immune organoids can provide early warning signals of potential CRS risk through cytokine profiling, they should be regarded as complementary to, rather than replacements for, systemic evaluation models when assessing severe immune-related adverse events. This platform greatly improves the human relevance of efficacy and toxicity assessment, and provides solid support for the early screening and mechanistic investigation of multi-specific antibodies.

A growing number of studies have adopted tumor immune organoids for the functional validation of bispecific and trispecific antibodies. The Roche research team established a co-culture system of patient-matched normal intestinal organoids and tumor organoids, which successfully recapitulated the specific off-tumor damage to human intestinal tissues caused by CEA- and EpCAM-targeted BiTEs, addressing the evaluation limitations of animal models (133). For BiTEs against solid tumors, another group developed gastric cancer organoid model co-cultured with PBMCs. They demonstrated that this system could accurately predict patients’ therapeutic responses to HER2×CD3 BiTE, and identified the lack of T cell help as a novel resistance mechanism to anti-HER2 mAb in gastric cancer (135). Furthermore, other researchers validated via healthy jejunal organoids co-cultured with human PBMCs that the affinity-attenuated CD3 binders decreased cytokine secretion with minimal on-target, off-tumor effects (136). Therefore, with the advantages of humanization and high simulation fidelity, tumor immune organoids effectively compensate for the deficiencies of traditional models and have become a core tool for investigating the preclinical efficacy, toxicity and mechanisms of action of bispecific and trispecific antibodies. Nevertheless, the integration of organoid-derived data with complementary approaches such as computational modeling, multi-organ-on-chip systems, or humanized mouse models may be necessary to comprehensively assess the risk of severe systemic toxicities prior to clinical translation (**Table 2**).

### 3.3 Tumor organoid platforms to develop combined regimens and reverse therapeutic resistance

Tumor organoids are powerful tools to dissect mechanisms underlying therapeutic resistance, while also enabling assessment of synergistic combinatorial regimens to reverse drug insensitivity. While immune checkpoint inhibitors have yielded impressive outcomes in clinical oncology, the ubiquitous phenomenon of therapeutic resistance remains a formidable bottleneck impeding sustained clinical benefits.

To address this challenge, patient derived organoids have emerged as a robust platform for developing novel strategies to circumvent resistance. Utilizing these patient-derived systems, Sun and colleagues investigated the therapeutic synergy of targeting TANK-binding kinase 1 (TBK1)-a pivotal kinase in innate immunity-in tandem with PD-1 blockade (137). Their findings demonstrated that the pharmacological or genetic modulation of TBK1 activity successfully abrogated the immune resistant phenotype of the tumor, validating TBK1 as a promising and novel target strategy to optimize immunotherapeutic efficacy (137). In research focusing on EGFR pathway-independent colorectal carcinoma, Mantilla-Rojas and colleagues validated that IL-10 signaling acts as a primary driver of tumor immune evasion (138). This finding supports the combinatorial use of EGFR-targeted agents and IL-10 neutralizing antibodies to robustly restore tumor susceptibility to treatment (138). Using human and mouse tumor organoid *ex vivo* models that recapitulate intact immune microenvironments, this study verified that high CD38 expression in exhausted CD8^+^T cells is a key driver of resistance to PD-1 immunotherapy in melanoma (139). Targeted CD38 inhibition improves T cell bioenergetics and restores antitumor effector functions, markedly resensitizing resistant tumors to anti-PD-1 antibodies, indicating CD38 as a promising combinatorial target to overcome PD-1 immunotherapy resistance (139). While organoid studies have provided mechanistic insights into TBK1-mediated resistance, the translation of TBK1 inhibitors into clinical trials remains an area of active investigation (**Table 2**).

Accumulating evidence has validated tumor organoids as a powerful platform to dissect resistance circuits of immunotherapy, screen optimal combination regimens and ultimately boost clinical benefits for patients. As an *in vitro* system capable of recapitulating patient unique tumor microenvironments and genomic profiles, organoids greatly strengthen the credibility of preclinical drug evaluation, facilitate the discovery of clinically actionable immunotherapeutic targets, and prevent underperforming agents from advancing to clinical trials. This organoid-based system expedites the clinical translation of personalized immunotherapies, cuts down research timelines and financial expenditures, and further refines treatment regimens for cancer patients.

### 3.4 Deciphering mechanisms underlying tumor-immune crosstalk

The heterogeneity of the tumor microenvironment is mainly determined by the population of stromal cells and immune cells. Moreover, the different functional states of these cells can be effectively simulated through patient derived tumor organoids. Analyzing the complex regulatory network formed by multiple cellular signaling pathways and cytokines is crucial for revealing the underlying mechanisms behind tumor immunotherapy. The cytokine interaction network regulates the dynamic balance of immune homeostasis in the tumor microenvironment. In this context, the *in vitro* co-culture model combining tumor organoids and immune cells has become an indispensable core research platform in this field.

In the TME, macrophages do more than phagocytose. Polarized into M1 or M2 phenotypes, TAMs exhibit pro-inflammatory or immunosuppressive traits, directly influencing tumor progression and treatment response (140). Multiple studies on organ co-culture have shown that factors secreted by tumor cells can promote the infiltration of TAMs and drive their migration towards M2 type. The resulting immunosuppressive state weakens the cytotoxic effect of anti-tumor cells, while also promoting tumor invasion and drug resistance formation (141). Interventions targeting related signaling pathways, such as inhibiting the cyclooxygenase-2 (COX-2)/prostaglandin E2 axis, can to some extent interrupt this immunosuppressive cell communication (142).

The impact of endothelial cells on the tumor microenvironment is not limited to the formation of blood vessels. It can participate in microenvironment regulation through vascular secretion signals and further act on immune cells. Research based on organoids has found that endothelial cells can affect macrophage polarization, tissue inflammation, and tumor metastasis processes in liver cancer, brain tumors, and ovarian cancer (143–145). However, the phenotype of commonly used endothelial cell lines and *in situ* endothelial cells within tumor tissue is not completely consistent. To address this limitation, human induced pluripotent stem cell-derived endothelial cells (iPSC-ECs) represent a promising alternative, as they can more faithfully recapitulate the molecular and functional properties of native tumor-associated endothelial cells while maintaining reproducibility and scalability (146). The integration of iPSC-ECs into organoid-immune cell co-culture systems may therefore provide a more physiologically relevant platform for studying vascular-immune interactions and precancerous microenvironment dynamics, thereby facilitating the optimization of existing models (147).

CAFs are also important stromal cells that determine the immune characteristics of the tumor microenvironment. Organoid studies have shown that CAFs have strong phenotypic plasticity, with transforming growth factor-β (TGF-β) and interleukin-1 (IL-1) inducing the formation of myofibroblast like CAF (myCAF) and inflammatory CAF (iCAF), respectively (145). Different CAF subgroups can shape immune inflammatory or immune rejection microenvironments, leading to differences in tumor malignancy and clinical prognosis (148). In various tumors, the paracrine signals released by CAFs can support tumor growth and enhance its tolerance to chemotherapy and immunotherapy (149–151). Due to the phenotype drift of primary CAFs in conventional culture, further improvements in organoid culture conditions are needed to more accurately analyze the mechanism of CAFs’ involvement in immune regulation and provide a more reliable model for the study of combination therapy regimens. However, due to the phenotypic drift of primary CAFs in conventional culture conditions, maintaining the distinct characteristics of iCAF and myCAF subpopulations during long-term organoid co-culture remains a significant unresolved technical challenge (152). This limitation hampers accurate mechanistic studies of CAF-mediated immune regulation and restricts the reliability of organoid-based models for screening combination therapy regimens. Further technological innovations in culture systems are urgently needed to preserve CAF heterogeneity and functional fidelity in long-term organoid platforms (153) (**Table 2**).

## 4 Current limitations and future prospective

Compared with traditional cell and animal models, patient derived tumor immune organoids have shown significant improvements in simulating the human tumor immune microenvironment, but existing co-culture systems still struggle to fully present the immune ecology in real tumor tissues. Most models only introduce a few immune components such as T cells or NK cells. While the infiltration and synergistic effects of MDSCs, TAMs, Tregs, and dendritic cells are often not fully reflected. In addition to incomplete immune cell composition, the reconstruction of matrix components such as CAFs, vascular endothelial cells, and extracellular matrix remodeling structures is also relatively limited. Due to the incomplete preservation of the immunosuppressive network and matrix barrier, existing models are difficult to accurately reproduce the complex immune escape process in solid tumors. As a result, the drug resistance of multi-specific antibodies, CAR-T, and NK cell immunotherapy in the real tumor microenvironment is difficult to fully simulate, which limits the in-depth study of drug resistance mechanisms and accurate judgment of clinical efficacy.

The lack of unified and standardized construction and evaluation protocols is one of the major bottlenecks limiting the widespread application of tumor immune organoids in immunotherapy drug evaluation. At present, significant differences exist across different research teams in terms of tumor organoid culture conditions, matrix gel types, immune cell sources, effector-target cell ratios, co-culture cycles, and drug intervention concentrations. Furthermore, there is no unified industry standard for detection indicators and evaluation systems for immunotherapy efficacy, including immune cell infiltration degree, cytotoxicity quantification, cytokine release level, and immune cell exhaustion degree. The inconsistent experimental systems and evaluation criteria result in poor repeatability and comparability of experimental data from different studies. It also hinders the establishment of unified evaluation standards for bispecific antibodies, trispecific antibodies, CAR-T and NK cell-based immunotherapies, limiting the clinical transformation and large-scale preclinical screening application of this model.

Sustained survival and stable functional maintenance of exogenous immune cells in tumor organoid co-culture systems remain a key technical difficulty. *In vitro* culture environments lack the *in vivo* cytokine supply, nutritional support and dynamic immune microenvironment regulation, resulting in rapid functional decline, exhaustion and apoptosis of co-cultured T cells, NK cells and other immune effector cells. Most current immune organoid systems can only support short-term co-culture within 1–2 weeks, which is difficult to meet the requirements for long term dynamic observation of immunotherapy effects, continuous monitoring of immune cell exhaustion, and exploration of chronic drug resistance mechanisms. To extend the operational window of tumor-immune organoids, continuous-perfusion bioreactors can improve nutrient delivery and waste removal, while cytokines such as IL-2 or IL-15 support T-cell and NK-cell survival and M-CSF or GM-CSF maintain myeloid populations. Selected studies have extended immune-cell viability to approximately 3–4 weeks. However, prolonged cytokine exposure may promote terminal differentiation, exhaustion-marker expression, and phenotypic divergence from the physiological state. Bioreactor systems also require specialized equipment and empirical optimization of cytokine concentrations, perfusion rates, and medium-exchange schedules, limiting standardization and scalability. Importantly, these approaches have not yet achieved the month-long culture periods needed to model durable immune memory, exhaustion reversal, or chronic therapeutic resistance. Thus, perfusion and cytokine supplementation provide partial improvements but do not fully overcome the limitations of immune-cell maintenance in organoid cultures. The inability to achieve long-term stable maintenance of immune cells severely restricts the evaluation of long-term anti-tumor efficacy and durable immune memory responses of novel immunotherapeutic agents such as trispecific antibodies and CAR-NK cells.

High culture costs and limited experimental scalability are important factors restricting the popularization and industrial application of tumor immune organoid models. The culture of patient derived tumor organoids relies on expensive matrix gels, specialized culture media and growth factors, and the separation and expansion of primary autologous immune cells further increase experimental costs and technical thresholds. Meanwhile, the traditional manual organoid culture method is difficult to achieve high-throughput and standardized batch preparation, which cannot meet the large-scale screening requirements of new immunotherapy drugs including bispecific/trispecific antibodies and cellular immunotherapies. In addition, the individual differences in patient derived samples lead to unstable model success rates, further increasing experimental time costs and limiting the application of tumor immune organoids in high-throughput drug screening and large-scale preclinical efficacy verification.

## 5 Future perspectives

### 5.1 Artificial intelligence-assisted organoid analysis

With the rapid development of artificial intelligence (AI) and digital pathology, AI-assisted image recognition and quantitative analysis have emerged as powerful tools to solve the quantification and standardization bottlenecks of tumor immune organoid research. Traditional manual observation and statistical methods are susceptible to subjective factors and have low efficiency, making it difficult to accurately quantify subtle changes in organoid morphology, immune cell infiltration density, and tumor cell apoptosis. Advanced artificial intelligence algorithms, including deep learning and machine learning models, can automatically identify the boundaries of organoids, quantify the depth and distribution patterns of immune cell infiltration, and dynamically calculate tumor cell survival rates and cytotoxicity efficiency. Importantly, AI models can integrate multidimensional data inputs from single-cell transcriptomics, spatial omics, proteomic profiling, and live-cell imaging to construct predictive models for immunotherapy response and drug resistance mechanisms. By learning complex patterns from high-dimensional omics datasets, AI-driven analytics enable the identification of subtle biomarkers and cellular interaction networks that would otherwise remain undetectable through conventional analysis. This synergy between omics data generation and AI-powered interpretation significantly enhances the objectivity, accuracy, and high-throughput analysis capabilities of organoid evaluation, providing a unified quantitative standard for multi-specific antibody detection, cellular immunotherapy, and vaccine efficacy evaluation, and promoting the standardization and intelligent development of preclinical organoid evaluation systems.

### 5.2 Single-cell multi-omics integration

Combining tumor immune organ models with single-cell multi omics and spatial omics techniques will greatly deepen the exploration of tumor immune interactions and immune therapy resistance mechanisms. Traditional batch detection methods can only reflect the average level of sample changes and cannot reflect the cellular heterogeneity and spatial specificity in the tumor immune microenvironment. By integrating single-cell transcriptomics, proteomics, and epigenomics techniques, multiple cell subpopulations, dynamic cellular state transitions, and key molecular changes in immune cell activation, depletion, and tumor immune escape processes can be identified with high resolution. By combining spatial transcriptomics and live cell imaging techniques, multi omics strategies can accurately map the spatial distribution of immune cells and stromal cells, reveal the molecular regulatory mechanisms of spatial immune rejection and microenvironment mediated drug resistance, and discover new immunotherapy targets and resistance biomarkers. Furthermore, multi-omics datasets serve as essential inputs for machine learning models, enabling the integration of transcriptomic, proteomic, and imaging features into unified predictive frameworks that can forecast therapy response, identify resistance signatures, and guide personalized treatment strategies. This integrated technology system bridges the gap between phenotype observation and molecular mechanism research, providing in-depth theoretical support for optimizing immunotherapy regimens and overcoming clinical drug resistance.

### 5.3 CRISPR-engineered organoids

CRISPR-Cas9 gene engineering technology has achieved precise gene editing of tumor immune organoids, targeted modification of key genes in tumor progression and immune regulation, greatly expanding the functional research value of organoid models. Through targeted knockout or overexpression of immune checkpoint genes, oncogenes or drug resistance-related genes, CRISPR-engineered organoids can simulate the occurrence process of tumor immune escape and acquired drug resistance, and verify the functional mechanism of key regulatory genes in immunotherapy response. In addition, gene-edited organoids can be used to screen and validate novel immunotherapeutic targets, optimize the design structure of bispecific/trispecific antibodies, and improve the tumor targeting and immune activation efficiency of CAR-T and CAR-NK cells. Moreover, CRISPR-based high-throughput gene screening combined with organoid platforms can systematically identify potential drug resistance genes and synergistic therapeutic targets, providing a reliable technical means for the development of new immunotherapy drugs and the optimization of combination treatment strategies.

### 5.4 Enhancing physiological relevance through microenvironment engineering

To improve the physiological relevance of tumor immune organoids, future efforts must focus on incorporating additional components of the tumor microenvironment that more comprehensively recapitulate *in vivo* complexity. Current organoid systems often lack critical stromal elements such as lymphatic endothelial cells, nerve fibers, adipocytes, and ECM diversity, all of which play essential roles in modulating immune responses and tumor progression. Integration of patient-derived CAFs, TAMs, and vascular networks into organoid cultures can better simulate the immunosuppressive landscape and metabolic stress conditions characteristic of solid tumors. Advanced bioengineering approaches, including 3D bioprinting, microfluidic organ-on-chip systems, and scaffold-based co-cultures, offer promising avenues to reconstruct spatially organized microenvironments with physiological stiffness, oxygen gradients, and perfusion dynamics. Moreover, ensuring reproducibility and scalability remains a critical challenge for translating organoid platforms beyond the laboratory. Standardized protocols for organoid initiation, expansion, cryopreservation, and quality control must be established to minimize batch-to-batch variability. Tailoring organoid models to specific research questions-such as evaluating checkpoint inhibitors, CAR-T cell infiltration, or vaccine-induced immunity-requires modular design strategies that allow flexible incorporation of relevant immune cell subsets and stromal components. The development of organoid biobanks with annotated clinical metadata will further facilitate reproducible, high-throughput screening and accelerate clinical translation.

### 5.5 Personalized precision immuno-oncology

Benefiting from high humanization and individualization, tumor immune organoid platforms are poised to become core carriers of future personalized precision tumor immunotherapy. Patient-derived tumor immune organoids completely retain individual tumor genetic characteristics, antigen profiles and unique immune microenvironment features, which can truly reflect patient-specific immunotherapy responses and drug resistance characteristics. In the future, organoid-based personalized evaluation systems can be applied to clinical patient stratification, enabling preclinical screening of optimal immunotherapy regimens including immune checkpoint inhibitors, multi-specific antibodies, cellular immunotherapy and tumor vaccines for individual patients. This model can effectively avoid ineffective treatment and reduce adverse immune reactions, such as CRS and off-tumor toxicity. Combined with clinical patient information and multi-omics data, organoid precision evaluation will help establish a whole-process predictive system for immunotherapy efficacy and safety, realize the transformation from empirical treatment to individualized precision treatment, and continuously promote the clinical popularization and precise application of tumor immunotherapy.

However, prospective clinical validation of organoid-based immunotherapy prediction remains limited. Although early studies have demonstrated the feasibility of using patient-derived organoids and autologous immune cells to assess treatment responses and tumor-reactive T-cell activity, most studies have involved small, single-center cohorts and have not yet established validated clinical prediction models. Current assays generally evaluate short-term *ex vivo* responses over approximately 1–2 weeks and cannot fully recapitulate systemic factors or long-term immune dynamics, including immune-cell trafficking, clonal evolution, and epitope spreading. In addition, the lack of standardized protocols and predefined clinical endpoints limits interlaboratory reproducibility and clinical implementation. Larger multicenter prospective studies with standardized procedures, independent validation, and long-term clinical follow-up are therefore required to determine whether organoid-based assays can reliably predict durable patient benefit.

## 6 Conclusion

The convergence of organoid biotechnology, systems immunology, spatial omics, and artificial intelligence is expected to redefine the future landscape of precision cancer immunotherapy. Traditional preclinical models have long restricted the translational progress of immunotherapy due to inherent limitations in simulating human tumor immune microenvironments. Tumor immune organoids, as innovative three-dimensional humanized models, effectively compensate for the defects of conventional systems, and have achieved remarkable advantages in evaluating the efficacy and safety of various immunotherapies, dissecting tumor-immune dynamic crosstalk, and exploring drug resistance mechanisms. Despite bottlenecks such as incomplete microenvironment reconstruction, insufficient standardization, unstable long-term immune cell maintenance, and high application costs, continuous innovation in cultivation techniques, intelligent analysis tools, and genetic engineering strategies will further optimize the fidelity and scalability of organoid models. In the future, the comprehensive application of artificial intelligence, multi omics, and CRISPR engineering will further unleash the potential of tumor immune organoids in basic research and clinical translation. Ultimately, this advanced preclinical platform will strongly support the development of personalized and precise immune ecology, accelerate the transition of new cancer immunotherapy from desktop to bedside, and bring more clinical benefits to cancer patients.
